# Isolation and Characterization of Novel Microsatellite Markers in Pomegranate (*Punica granatum* L.)

**DOI:** 10.3390/ijms11052010

**Published:** 2010-05-03

**Authors:** Seyed Mostafa Pirseyedi, Sahar Valizadehghan, Mohsen Mardi, Mohammad Reza Ghaffari, Parvaneh Mahmoodi, Mehdi Zahravi, Mehrshad Zeinalabedini, Seyed Mojtaba Khayam Nekoui

**Affiliations:** 1 Department of Genomics, Agricultural Biotechnology Research Institute of Iran, Mahdasht Road, Karaj, Iran; E-Mails: m_pirseyedi@abrii.ac.ir (S.M.P.); s.valizadegan@gmail.com (S.V.); ghaffary@abrii.ac.ir (M.R.G.); mparvaneh59@yahoo.com (P.M.); mzeinolabedini@abrii.ac.ir (M..Z.); khayam@abrii.ac.ir (S.M.K.N.); 2 Iranain National Gene Bank, Mahdasht Road, Karaj, Iran; E-Mail: mzahravi@yahoo.com

**Keywords:** *Punica granatum*, microsatellite, pomegranate, SSR

## Abstract

Pomegranate (*Punica granatum* L.) has been cultivated from ancient times for its economic, ornamental and medicinal properties globally. Here, we report the isolation and characterization of 12 polymorphic microsatellite markers from a repeat-enriched genomic library of *Punica granatum* L. The genetic diversity of these loci was assessed in 60 genotypes of *Punica granatum* L. All loci were variable: the number of polymorphic alleles per locus ranged from two to five (average 2.9). The observed and expected heterozygosities ranged from 0.15 to 0.87 and 0.29 to 0.65, respectively. The polymorphic information content ranged from 0.26 to 0.61 (average: 0.43). To the best of our knowledge, this is the first time that polymorphic microsatellite markers have been reported for *P. granatum* L. These new markers should allow studies of the population structure and genetic diversity of pomegranate to be performed in the future.

## 1. Introduction

The pomegranate (*Punica granatum* L.) probably originated in Iran [1], and from there diversified to other regions such as the Mediterranean. Large areas of Iran within the boundaries of the two deserts that occupy the central Iranian plateau (Dasht-e-kavir and Kavir-e-Loot) have arid or semiarid conditions that make them suitable for pomegranate production. In fact, the pomegranate has been cultivated from ancient times for its economic, ornamental and medicinal properties in all of the provinces that border the central desert. In these provinces, the area under cultivation, rate of expansion, diversity of varietals, yield per tree, and quality of the product is all considerable. All of these factors support the fact that the pomegranate is endemic to Iran [2].

To understand the structure of the population, to prevent duplication, and assess the variation of this valuable species accurately, it is necessary to characterize each accession not only in terms of its morphological variation, but also by a genome-wide survey of genetic diversity. Although a wide range of morphological and physiological characteristics show variability in the pomegranate, only a few studies based on molecular markers have been performed to investigate the population dynamics of this economically important species [3–7]. Here, we report the isolation and characterization of the first polymorphic microsatellite markers for pomegranate.

## 2. Results and Discussion

Out of 80 clones sequenced, it was possible to design unique primers for 58 (72%). For the remaining clones, in some cases, the sequence quality was poor and in others, the SSRs were too close to the start or end of the insert. The polymorphism of the SSR markers was examined in 60 samples of *P. granatum* L. Twelve of the 58 markers were scorable and polymorphic. For these 12 markers, 35 alleles were identified (Table 1); the number of alleles ranged from two to five, with an average of 2.9 alleles per locus. The observed (Ho) and expected (He) heterozygosities ranged from 0.15 to 0.87 and 0.29 to 0.65, respectively. The PIC values ranged from 0.26 to 0.61, with an average of 0.43. Out of 12 polymorphic loci, 10 were departed significantly from the Hardy-Weinberg equilibrium (HWE) (P < 0.05).

In this study, 12 polymorphic microsatellite markers for pomegranate were developed from an enriched partial genomic library that was constructed using the fast isolation by AFLP of sequences containing repeats (FIASCO) protocol. The efficiency of this method in this species was approximately 73%, which conformed to the expected percentage of efficiency reported [8]. In our sample, some markers deviated from the Hardy-Weinberg equilibrium. This result could be due to loss of genetic diversity in small random mating populations, small numbers of microsatellite markers, possible group structures and a mating system with a high level of inbreeding. A larger number of markers would still be required in future to enable wider genome coverage.

To the best of our knowledge, this is the first time that polymorphic microsatellite markers have been reported for *P. granatum* L. Iran hosts a great genetic diversity of *Punica granatum* and more than 760 Iranian genotypes are collected at Iranian national pomegranate in Yazd, Iran. However, the study of genetic diversity, genetic background and mating behavior in Iranian pomegranate has been limited because of the lack of sufficient polymorphic microsatellite markers.

## 3. Experimental Section

### 3.1. Isolation of SSR Markers

Genomic DNA was extracted from a pomegranate cultivar “Malas Yazd” using DNeasy Plant Mini kit (Qiagen, Germany). A genomic library enriched for di- and trinucleotides was constructed using the fast isolation by AFLP of sequences containing repeats (FIASCO) protocol [8,9] with some modifications. A total of 250 ng genomic DNA was digested with *Mse*I (Fermentase) to give DNA fragments between 200 and 1000 bp in length. The fragments were ligated to adapters and then amplified in two stages by PCR using *Mse*I primers to give numerous copies of each fragment. The genomic DNA fragments that contained SSRs were captured by hybridization to biotinylated probes that consisted of di- and trinucleotide repeats [(GC)_17_, (AC)_17_, (CT)_17_, (AT)_17_, (GT)_17_, (ATT)_10_, and (CTT)_10_], followed by binding to streptavidin-conjugated magnetic beads (BioMag®; Qiagen, Germany). Three non-stringent and three stringent washes were carried out with separation in a magnetic field. The recovered DNA fragments were amplified for 30 cycles using the *Mse*I primers. The PCR products were cloned into pGEM-T Easy (Promega, Germany), and transformed into *Escherichia coli* DH5*a.* Recombinant clones were identified by blue/white screening and restriction analysis (Eco*RI*; Fermentase, Germany). Eighty clones with inserts were purified using a plasmid extraction kit (Core-Bio, Canada) and sequenced (Macrogen Sequencing Service, Korea). Fifty eight of the clones contained microsatellite repeats and it was possible to design primers for them.

PCR amplification was performed on an ABI thermal cycler in a total volume of 15 μL, which included 20 ng DNA, 1× PCR buffer, 2 mM MgCl_2_, 0.06 pmol each primer, and 0.5 U Taq DNA polymerase (Fermentase, Germany). The following reaction conditions were used: 5 min at 95 °C, followed by 10 touchdown cycles of 30 s at 95 °C, 45 s at 60 °C (1 °C lower per cycle) and 40 s at 72 °C, and 25 cycles of 30 s at 95 °C, 30 s at 50 °C and 40 s at 72 °C, with a final extension step of 7 min at 72 °C. Amplified products were separated on 6% denaturing polyacrylamide gels and visualized by silver staining. A 50-bp DNA ladder (Fermentase, Germany) was used to identify the alleles.

### 3.2. Data Analysis

The variability of these markers was analysed in 60 *Punica granatum* L. genotypes that were sampled from Iranian national pomegranate collection, Yazd, Iran (Table 2). POPGENE 32 [10] was used to calculate the observed and expected heterozygosities and to evaluate deviation from Hardy–Weinberg equilibrium (HWE) and linkage disequilibrium between pairs of loci. All results were adjusted for multiple simultaneous comparisons using a sequential Bonferroni correction [11]. Polymorphic information content (PIC) was estimated using CERVUS v.2.0 [12].

## 4. Conclusions

Pomegranate germplasm collections will be benefited by utilizing the isolated microsatellite markers. These markers can complement morphological and pomological traits analyses for accurate population genetics studies and assessing genetic variations. They are also expected to be useful for efficient genetic studies, e.g., linkage analysis, construction of molecular linkage maps and marker-assisted breeding on *Punica granatum* L. These polymorphic microsatellite markers would be useful tools for future collection strategies and management of pomegranate genetic resources.

## Figures and Tables

**Table 1.** Characterization of 12 polymorphic microsatellite loci in pomegranate (*Punica granatum* L.).

**Locus**	**Primer sequences (5′→3′)**	**Repeat motif**	**Expected product Length/observed range (bp)**	**No. of alleles**	***H_O_***	***H_E_***	**PIC**	**Accession Number**
ABRII-MP04	F:5-CAGGTGATTGACTACTTGG-3	(GT)_7_	201/195–215	2	0.82**	0.48	0.36	
	R:5-CAGATCTACAATAACATCAC-3							GU950619
ABRII-MP07	F:5-GATTAACAGCAAAGCCTAGAGG-3	(AT)_9_(GT)_7_	181/180–190	2	0.39	0.32	0.39	
	R:5-AGTAGCTGCAACAAGATAAGG-3							GU950620
ABRII-MP12	F:5-TTGAGTCCCGATCATATCTC-3	(CA)_11_	270/240–270	3	0.60**	0.42	0.34	
	R:5-TCAATCTGTCAGGAACAACA-3							GU950621
ABRII-MP26	F:5-TTTCTCGAAGAATTGGGTAA-3	(AG)_25_	166/145–160	5	0.37**	0.62	0.60	
	R:5-CTGAGTAAGCTGAGGCTGAT-3							GU950622
ABRII-MP28	F:5-ATCCTCTGTCTTTGTGTTCG-3	(GAGG)_3_(GA)_19_	349/350–390	3	0.15**	0.65	0.58	
	R:5-TGAGTAATTCCGGTCAGAAG-3							GU950623
ABRII-MP30	F:5-CCCAGTTTGTAGCAAGGTA-3	(TGAGC)_3_	175/160–190	3	0.85**	0.60	0.52	
	R:5-AAGCTGACATTCTTTGAAGC-3							GU950624
ABRII-MP33	F:5- TCTGTTTATTGCTGAAAGGG-3	(AG)_12_	105/80–120	2	0.65**	0.43	0.34	
	R:5-TCTTCTTCTTCTCCACCGTA-3							GU950625
ABRII-MP34	F:5-GGAAGAAGCAGAGCAATAGA-3	(GAA)_3_	210/180–220	3	0.75**	0.48	0.36	
	R:5-GTCCTGAGTAACCTGAGCTG							GU950626
ABRII-MP39	F:5-AGTCTCTGAAGTTTGTCGGA-3	(GA)_8_(TTTTCT)_2_	252/250–305	2	0.30	0.29	0.26	
	R:5-CCTGAGTAAAGCATCTCACTG-3							GU950627
ABRII-MP42	F:5-GAGCAGAGCAATTCAATCTC-3	(GA)_9_	194/200–220	4	0.87**	0.61	0.54	
	R:5-AACAATTTCCCATGTTTGAC-3							GU950628
ABRII-MP46	F:5-AGTTGATCTGATGGACAAGG-3	(GTT)_4_	270/250–300	2	0.51**	0.38	0.31	
	R:5-CAGTACGGTGCTCAATACAA-3							GU950629

H_O_, observed heterozygosity;

H_E_, expected heterozygosity;

** indicated deviation from Hardy–Weinberg equilibrium after Bonferroni correction *(P* < 0.01); PIC, polymorphic information content.

**Table 2.** Iranian pomegranate genotypes included in the study.

**Genotypes**	**Collection Label**	**Origin**
Torsh nar riz zirab	R2-69-2	Mazandaran
Meikhosh marvast mehriz	R3-67-23	Yazd
Shoor poost nazok saghand	R1-71-5	Yazd
Panje aroos khafr torh	R1-67-22	Fars
Ardestani malas darjzin	R1-69-3	Semnan
Shirin dane sefid mehran	R2-71-28	Ilam
Shahvar shirin	R4-68-29	Yazd
Amene khatooni abrandabad malas	R6-67-1	Yazd
Poost sefid paveh torsh	R1-70-5	Kordestan
Torsh ashraf	R2-70-30	Mazandaran
Shirin ardestan	R4-70-14	Isfahan
Togh gardan	R2-70-23	Yazd
Torsh poost nazok	R4-70-24	Chahar mahal bakhtiari
Torsh poost sefid tarom	R5-70-5	Zanjan
Tabestani save	R5-70-16	Markazi
Vahshi tamini torsh	R5-70-21	Sistan baloochestan
Jazi poost ghermez shirin	R5-68-9	Kerman
Malas yazdi	R1-67-14	Yazd
Shririn jazireh	R2-69-3	Booshehr
Shirin poost nazok darjzin	R2-69-4	Semnan
Siahdane shahvarkan malas	R2-69-11	Tehran
Malas dane siah ramhormoz	R2-69-13	Khoozestan
Vahshi jangali babolsar torsh	R2-69-21	Mazandaran
Shirin poost sefid shahreza	R2-69-30	Isfahan
Mesri torsh kazeroon	R2-69-7	Fars
Garach shahvar yazdi	R1-67-21	Yazd
Malas soorati dezfool	R4-69-19	Khoozestan
Tolfdar dane ghermez malas	R4-69-25	Khoozestan
Bitolf dane ghermez malas	R4-69-29	Khoozestan
Vahshi jangali ghaemshar	R5-69-2	Mazandaran
Sorkhpoost ize	R5-69-3	Khoozestan
Narak pishva varamin	R5-69-20	Tehran
Vahshi jangali roodsar	R5-69-26	Tehran
Malas zoodras kan	R4-69-10	Tehran
Zardanar poost nazok	R5-67-2	Fars
Rash poost nazok	R6-69-18	Kohkilooye
Zagh yazdi	R1-67-3	Yazd
Malasie bidane malas	R6-69-20	Semnan
Shiri ahmadi gogrgan	R6-69-21	Mazandaran
Goli zirab savadkooh torsh	R6-69-23	Mazandaran
Shirin taghlid kon	R4-69-28	Tehran
Dombkooh sarjangal shirin	R5-67-16	Kerman
Bihaste sangan shirin sistan	R3-67-21	Sistan baloochestan
Meykhosh poostnazok	R1-67-26	Kerman
Khajeatar mehriz malas	R5-68-2	Yazd
Soghar naiin torsh	R5-67-1	Isfahan
Malas soori paveh	R5-70-15	Kermanshah
Vashik torsh	R6-67-30	Sistan baloochestan
Vahshinarak torsh	R1-67-13	Fars
Shoorporbar seidoon torsh	R3-67-22	Fars
Poost sefid abrandabad	R4-67-15	Yazd
Gol peivandi torsh	R3-68-29	Yazd
Domboland tosh	R5-69-15	Mazandaran
Aghamohseni shirin	R2-69-24	Mazandaran
Ghors galooboland bafgh malas	R5-70-4	Yazd
Ood poostghermez	R6-67-29	Fars
Asali sarvestan shirin	R5-67-9	Fars
Khafri jahrom shirin	R3-67-19	Fars
Bazri shirin dastjerd	R1-67-24	Isfahan
